# The influence of air pollutants on the risk of emergency department presentations of infants with bronchiolitis in an European air quality hotspot

**DOI:** 10.1111/pai.70077

**Published:** 2025-04-02

**Authors:** D. Zama, A. Paccapelo, L. Betti, E. Manieri, M. Paglione, M. Rinaldi, A. Dondi, E. Battelli, C. Biagi, C. Marchegiani Rizzolli, P. Manzoni, G. Piglia, G. Nicolini, M. Lanari, C. Carbone

**Affiliations:** ^1^ Department of Medical and Surgical Sciences, Alma Mater Studiorum University of Bologna Bologna Italy; ^2^ Pediatric Emergency Unit IRCCS Azienda Ospedaliero‐Universitaria di Bologna Bologna Italy; ^3^ Epidemiology and Statistics IRCCS Azienda Ospedaliero‐Universitaria di Bologna Bologna Italy; ^4^ Specialty School of Paediatrics, Alma Mater Studiorum University of Bologna Bologna Italy; ^5^ Italian National Research Council‐Institute of Atmospheric Sciences and Climate (CNR‐ISAC) Bologna Italy; ^6^ Pediatric Unit, Department of Women's and Children's Health University Hospital of Padua Padua Italy; ^7^ Department of Maternal‐Infant Medicine, “Degli Infermi” Hospital University of Torino School of Medicine Ponderano, Biella Italy; ^8^ Unità Operativa Complessa Pediatria San Martino Hospital Belluno Italy; ^9^ Italian National Agency for New Technologies, Energy and Sustainable Economic Development (ENEA) Bologna Italy

**Keywords:** bronchiolitis, carbonaceous fractions, children, hospitalization, infants, outdoor air pollution, particulate matter

## Abstract

**Background:**

Acute bronchiolitis is the leading cause of hospitalization in infants, and air pollutants represent a risk factor for its development. This work aims to investigate the role of air pollution, considering conventional and nonconventional indicators, in the development of bronchiolitis in three urban areas in the Po Valley, Northern Italy.

**Methods:**

This multicentric, observational, retrospective, cohort study included infants under 12 months who were referred to the Pediatric Emergency Department of Bologna, Belluno, and Biella and diagnosed with bronchiolitis from 2016 to 2019. Data on daily ground‐level mass concentrations of particulate matter (PM_10_ and PM_2.5_) and gaseous pollutants in the three areas, and additionally of organic carbon (OC) and elemental carbon (EC) in Bologna, were retrieved and assessed for possible relationships with the occurrence of bronchiolitis.

**Results:**

A total of 1316 patients were enrolled. All conventional air quality indicators (NO_2_ , PM_10_ , and PM_2.5_) showed statistically significant associations with the occurrence of referrals due to bronchiolitis. The highest impacts were observed for OC and EC, the carbonaceous components of PM, which were only measured in Bologna. Considering the conventional indicators, the strongest associations were found between 4‐week moving average concentrations and weekly hospital admission, and the strongest associations were found considering NO_2_ and PM_2.5_.

**Conclusion:**

This study indicates that medium‐term exposure to higher levels of air pollution increases the risk of the development of bronchiolitis. In particular, the best association results between bronchiolitis admissions and the exposure to the carbonaceous fraction of PM_2.5_.

**Abbreviations**
AIC: Akaike's information criterion
BIC: Bayesian information criterion
C_6_H_6_
: benzene
CI: confidence interval
CO: carbon monoxide
EC: elemental carbon
IRRs: incidence rate ratios
NO: NO_2_ nitrogen oxides
O3: ozone
OC: organic carbon
OR: odds ratio
PED: Pediatric Emergency Department
PM: particulate matter
PM10: particulate matter with an aerodynamic diameter of 10 μm
PM2.5: particulate matter with an aerodynamic diameter of 2.5 μm
RDAI: Respiratory Distress Assessment Instrument
ROS: reactive oxygen species
RSV: respiratory syncytial virus
RV: rhinovirus
SO: SO_2_ sulfur oxides
SOA: secondary organic aerosol
SSOU: Short‐Stay Observation Unit
WHO: World Health Organization

**Key Message**

This study, conducted in three urban areas in the Po Valley in Italy, reveals that exposure to high levels of air pollution increases the risk of bronchiolitis; in particular, with bronchiolitis admissions linked to exposure to the carbonaceous fraction of fine PM_2.5_. This finding indicates that the carbonaceous fraction may serve as a more accurate proxy for the associations between air pollution and bronchiolitis than the classical mass concentration. Children are particularly vulnerable to pollutants, and their exposure should be minimized in areas most affected by anthropogenic pollution sources through the implementation of mitigation policies.

## 1 INTRODUCTION

Bronchiolitis is the leading cause of respiratory tract infection and hospitalization in children younger than 1 year, caused by viruses, mainly respiratory syncytial virus (RSV) and Rhinovirus (RV).^1^, ^2^ The clinical spectrum of bronchiolitis ranges from mild infections to severe forms requiring hospitalization,^2^ including admission to intensive care units due to the need for invasive ventilation. The risk factors for severe forms include prematurity, chronic lung disease, congenital heart disease, neurological disease, and immunodeficiency.^3^ It is well established that bronchiolitis can predispose to subsequent wheezing episodes, whereas the link with the onset of asthma is unclear.^4^ Bronchiolitis presents a seasonal pattern, with the highest incidence in winter in temperate climates.^5^

Air pollution represents one of the leading environmental risks to human health. Exposure to air pollutants is ubiquitous, and according to the World Health Organization (WHO), around 93% of children breathe polluted air every day.^6^ Young children are particularly vulnerable to air pollution due to their developing respiratory and immune systems, lower oxidative stress response, higher breathing rates, and their proximity to ground‐level particulate matter (PM).^7^, ^8^

Air pollutants with the most significant negative effects on health include PM, classified into PM with a diameter smaller than 2.5 micrometers (PM_2.5_) or 10 micrometers (PM_10_), nitrogen dioxide (NO_2_), sulfur dioxide (SO_2_), carbon monoxide (CO), ozone (O_3_), and benzene (C_6_H_6_).^9^ Despite the various studies, the exact role of pollutants in the development and severity of bronchiolitis is unclear. Moreover, it is not yet clear which is the best methodology or the most appropriate type of study to understand these correlations.^10^

This work investigates the role of air pollution, considering conventional and nonconventional indicators, on the risk of acute bronchiolitis hospitalization in three urban areas in the Po Valley, a densely populated and industrialized region in Northern Italy, known as a European air quality hotspot.^11^ The frequent stability of atmospheric conditions and higher emissions primarily from residential heating, vehicular traffic, and biomass burning facilitates the accumulation of airborne pollutants, often on a large scale.^12^, ^13^

## 2 MATERIALS AND METHODS

### 2.1 Study design and setting

This is a multicentric, observational, retrospective, cohort study, including infants under 12 months referred for bronchiolitis from 1st October 2016 to 31st December 2019, in the Pediatric Emergency Department (PED) of three Italian centers: IRCCS‐AOUBO Policlinico of S.Orsola in Bologna, the “Nuovo Ospedale degli Infermi” in Ponderano (Biella) and the Hospital of S. Martino in Belluno. The IRCCS AOUBO in Bologna is a University tertiary‐care Pediatric Emergency Unit with a PED with an average of 24,000 visits/year, a 6‐bed short‐stay observation unit (SSOU), and a 24‐bed ward. A pediatric intensive care unit is present. The “Nuovo Ospedale degli infermi” in Ponderano (Biella) is a second‐level center, with 24 total beds including SSOU, and an average of 6000 emergency visits/year. The Hospital of S. Martino in Belluno is a second‐level center, with 15 beds, the SSOU, and an average of 5000 emergency visits/year.

**FIGURE 1:**
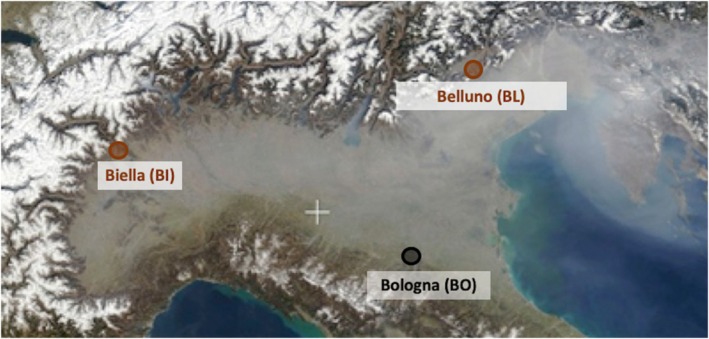
Map of the domain of this case study, Po Valley, Northern Italy.

The three centers are located at the boundaries of a hypothetical triangle covering much of the Po Valley in Northern Italy (Figure 1). Biella, with 44,000 residents, lies in the northeast (420 meters asl), near the Alps; Belluno, with 36,000 residents, is in the northwest (390 meters asl), near the Alpine Dolomites; Bologna, with 390,000 inhabitants (or 1 million, including the urban area), lies in the southern Po plain (50 meters asl), near the Apennines. These towns are similarly affected during the colder months by stable atmospheric patterns and emissions from residential heating and biomass burning that lead to severe air pollution episodes.^12^, ^13^ The three selected towns represent typical pollutant conditions for a highly industrialized, urbanized, and densely populated macro‐region in the Northern Hemisphere at mid‐latitudes.

The epidemiological season for bronchiolitis coincides with the winter months, while there is a significant decrease to a low number of cases during summer. This alignment in the annual trends creates a covariance between clinical data and air quality indicators, which is not necessarily causal. To eliminate this source of covariance, we restricted our analysis to the epidemic months, from the beginning of October to the end of March, when there is a greater probability that any observed correlations between air quality and clinical data are truly causal.

### 2.2 Clinical data collection and participants

For each patient, we considered the following data: demographic data, presence of known risk factors for the development of bronchiolitis or eating difficulties, type of feeding, previous hospital access for the same episode of bronchiolitis, vital signs at PED arrival, discharge modality, and complications (Table S1).

Based on the data collected, the severity index of bronchiolitis according to Baraldi et al.^14^—considering the respiratory rate, respiratory effort, oxygen saturations, feeding, and apnea— and the clinical score Respiratory Distress Assessment Instrument (RDAI) modified by Basile et al.^15^—considering the respiratory rate, the presence of distress, the use of accessory respiratory muscles, and the thoracic auscultation—were calculated. According to Italian guidelines,^14^, ^16^ hospitalization criteria include moderate‐to‐severe bronchiolitis, inability to maintain adequate hydration, O_2_ saturation levels below 92%, risk factors for severe bronchiolitis, and family reliability. Discharge criteria require independence from respiratory supports and adequate O_2_ saturation levels, stable clinical conditions, adequate oral intake of liquids and feed, and family capable of managing symptoms and therapy at home.

### 2.3 Air quality data

Daily ground‐level mass concentrations of PM_10_ and PM_2.5_ and gaseous NO_2_, as routinely measured by the air quality monitoring program of the Regional Environmental Protection Agencies (ARPAs), are considered. Measurements are performed according to the EU Directive 2008/50, following the international standards UNI EN ISO 12341:2023 and 14211:2025 for PM and NO_2_, respectively. PM mass and NO_2_ concentration data are all expressed as μg m^−3^. In line with the criteria set by the European Environment Agency, we selected background monitoring sites in the three study towns—Bologna, Biella, and Belluno—representative of population exposure at the urban scale, approximately corresponding to the exposure experienced by users of the hospitals included in the analysis.

In Bologna, additional chemical analyses of PM_2.5_ samples were conducted to measure concentrations of carbonaceous fractions, specifically organic carbon (OC) and elemental carbon (EC), which were quantified on quartz fiber filters using thermo‐optical transmission analysis (Sunset Laboratory Inc., Oregon, USA) following the EUSAAR2 thermal protocol.^17^, ^18^ Both OC and EC data are expressed in μg m^−3^. OC consists of organic chemical species originating from various anthropogenic and biogenic sources and can be (i) primary, that is, directly emitted from a variety of combustion sources, such as fossil fuel and biomass combustion from traffic and residential heating, which strongly impacts the wide area of the Po basin during colder months^19^; (ii) secondary, that is, formed in the atmosphere after undergoing oxidation from different sources (e.g., livestock/agriculture activities, vehicular traffic, residential heating with fossil fuels etc.). EC represents the portion of PM generated by the incomplete combustion of carbon‐based fuels (such as those from traffic and residential heating). It is characterized by a semi‐graphitic chemical structure and is frequently co‐emitted with primary OC. Given the limitations of conventional mass‐based metrics for PM,^20^, ^21^ incorporating these carbonaceous fractions aims to evaluate the relationship between specific chemical components and PM sources and the health outcomes being considered.

### 2.4 Statistical data analysis

Patient demographic and clinical characteristics were reported as frequencies and percentages for categorical variables and as mean ± standard deviation or median and range for continuous variables. The Spearman's rank correlation coefficients were computed for pollutants and visits, and the results were considered to select the best setting for multivariable analysis. Daily and weekly visits to the PED were used as dependent variables for the predictive models. They were computed for each day observed, considering the number of visits on the day or in the following week. We calculated the moving averages for all pollutants by adding the pollutant values for the last 7 or 28 days and dividing by the number of days. The 1‐week and 4‐week moving averages of pollutants were considered as predictors for all the models, matching the last day of average calculation with the first day of visits counting. The last day of the averaging period was aligned with the first day of visit counting to ensure a temporal correspondence between pollutant exposure and the analyzed visits. The Poisson generalized linear model was used. We also considered the linear regression, the zero‐inflated Poisson model, and the negative binomial regression but the Poisson model showed better fitting performance on our data. The incidence rate ratios (IRRs) were computed together with their 95% confidence intervals (95%CI). The scatter plot was used to graphically compare fitted values and observed values, and the *R* ^2^ for this evaluation was computed. The Akaike's information criterion (AIC), the Bayesian information criterion (BIC), the log‐likelihood, and McFadden's pseudo *R* ^2^ were computed for model comparison. Box plots and line plots with 95% CIs were also used to represent the data. The *p* value was considered significant when less than .05 for two‐tailed tests. Statistical analyses were performed using IBM SPSS Statistics for Windows software, Version 29.0 (Armonk, NY: IBM Corp).

## 3 RESULTS

### 3.1 Clinical population

From October 2016 to December 2019, in the epidemic months from the beginning of October to the end of March of each year, 1316 children were managed by the three PEDs for a diagnosis of bronchiolitis. Five hundred seventy‐four (43.6%) were discharged after being visited, 132 (10%) were kept in the SSOU, and 610 (46.4%) were admitted to the ward. RSV was tested in 732 patients, resulting positive in 409 patients (55.9%). The clinical and demographic characteristics of the total population divided into the three centers are described in Table 1.

**TABLE 1:** Clinical and demographic characteristics of the study population.

	Total	Bologna	Biella	Belluno
Patients, *n* (%)	1316 (100)	1078 (100)	161 (100)	77 (100)
Male, *n* (%)	776 (59)	650 (60.3)	87 (54)	39 (50.6)
Age mean ± SD, months	5.6 ± 3.5	6.2 ± 3.5	3.1 ± 2.3	4 ± 3
Weight ± SD, kg	6.9 ± 2.2	7.2 ± 2.1	5.2 ± 1.5	5.8 ± 2.8
Ethnicity, *n* (%)				
Caucasian	943 (71.7)	772 (71.6)	109 (67.7)	62 (80.5)
African	180 (13.7)	155 (14.4)	15 (9.3)	10 (13)
Asian	139 (10.6)	137 (12.7)	0	2 (2.6)
Not defined	54 (4)	14 (1.3)	37 (23)	3 (3.9)
Prematurity *n* (%)	112 (8.5)	85 (7.9)	19 (11.8)	8 (11.1)
GA <34 weeks *n* (%)	53 (47.3)	40	10	3
Risk factors^a^, *n* (%)	68 (5.2)	54 (5)	12 (7.5)	2 (2.6)
Feeding				
Breastfeeding	315 (48.2)	204 (19)	83 (51.6)	28 (36.4)
Mixed feeding	226 (34.6)	183 (17)	37 (23)	6 (7.8)
Formula feeding	113 (17.2)	76 (7.1)	27 (16.8)	10 (13)
Eating difficulties	685 (52.1)	518 (48)	128 (80)	39 (54.9)
Days from symptoms onset, mean ± SD	4 ± 4.3	4 ± 2.6	4 ± 2	4 ± 1.2
Previous access, *n* (%)	237 (18)	181 (16.8)	40 (24.8)	16 (20.8)
Severity^b^, *n* (%)				
Mild	946 (73.5)	837 (77.6)	55 (42)	54 (70.1)
Moderate	304 (23.6)	216 (20)	66 (50.4)	22 (28.6)
Severe	36 (2.9)	25 (2.3)	10 (7.6)	1 (1.3)
Clinical score^c^, *n* (%)				
Mild	1005 (78.5)	893 (82.8)	69 (50)	44 (66.7)
Moderate	263 (20.5)	179 (16.6)	62 (44.9)	22 (33.3)
Severe	13 (1)	6 (0.6)	7 (5.1)	0 (0)
Pneumonia, *n* (%)	134 (10.2)	114 (10.6)	11 (6.8)	9 (11.7)
Other complications^d^, *n* (%)	9 (0.7)	6 (0.6)	3 (1.9)	0 (0)

^a^ Risk factors: previous apnea, previous episodes of wheezing, chronic lung disease, congenital heart disease, immunodeficiency, and severe neurological or muscular pathology.

^b^ According to Baraldi et al.^14^

^c^ Modified by Basile et al.^15^

^d^ Other complications: sepsis, pneumothorax, and pleural effusion.

### 3.2 Air quality

The time‐series of concentrations for the main pollutants (Figures S1–S4) indicate that there is generally minimal inter‐annual variability in air quality indicators during the years analyzed. All the considered parameters show consistent minima and maxima (with few exceptions) in the different years and a seasonal evolution of the concentration levels peaking in the central months of the epidemic season in all three selected cities.

**FIGURE 2:**
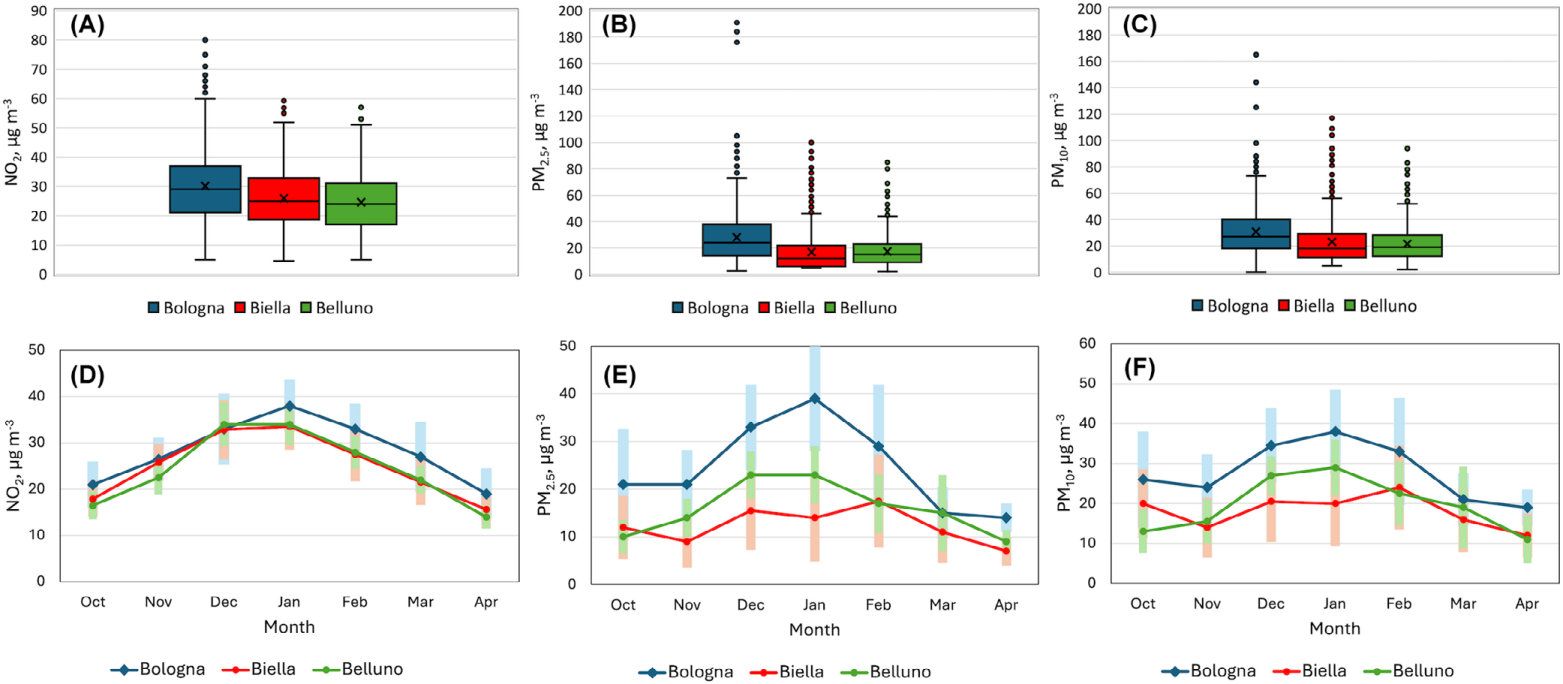
Box plots of daily data concentrations (panels A, B, and C) and monthly median and IQR (panels D, E, and F) of NO_2_, PM_2.5_, and PM_10_, based on the 2016–2019 period (from September to April).

The summary statistics for air quality indicators presented in Figure 2 (panels (A), (B), and (C)) indicate significantly higher levels of pollution in Bologna compared to Biella and Belluno, with all three indicators showing marked increases (*p* < .001). While Biella has higher NO_2_ levels than Belluno, it records lower PM levels. The differences between Biella and Belluno are statistically significant for all indicators except PM_2.5_.

Panels (D), (E), and (F) of Figure 2 illustrate the monthly medians of NO_2_, PM_10_, and PM_2.5_ during the colder months. The period of maximum atmospheric pollution spans from December to February in all three cities, with January typically being the peak month. In Biella, the peak for PM levels appears to shift to February.

### 3.3 Epidemiological analysis and predictive modeling

To assess the relationship between air pollution and the risk of hospitalization for bronchiolitis, we first conducted an exploratory analysis to examine the partial correlations between mean pollutant levels (using both 1‐week and 4‐week moving averages) and the number of visits (both daily and weekly). The strongest associations were found when considering the 4‐week moving average concentrations of pollutants in relation to weekly visits for bronchiolitis (Tables S2 and S3).

**FIGURE 3:**
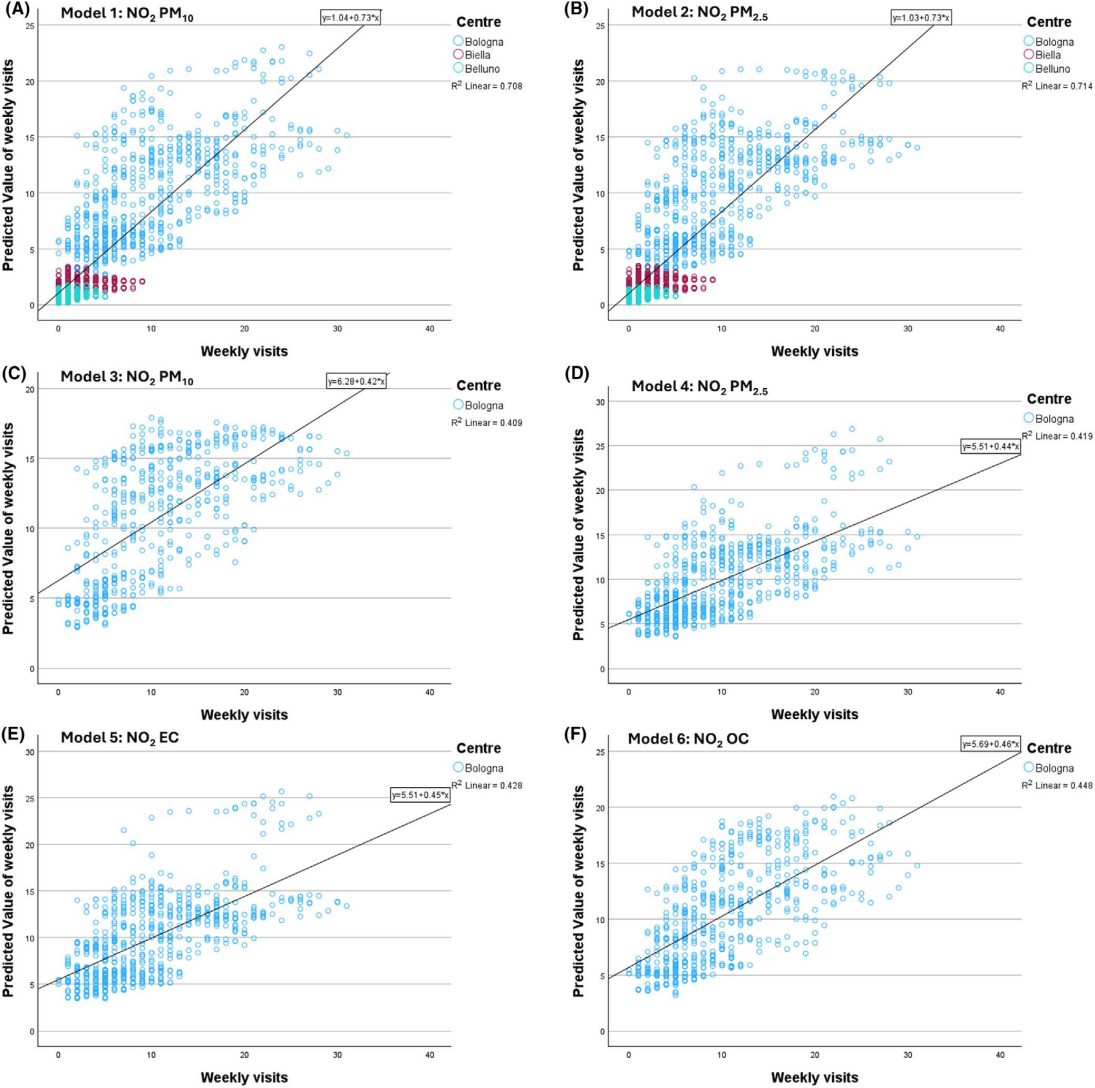
Model 1, 2, 3, 4, 5, and 6 outcomes. Panels (A) and (B) show the association between the conventional air quality indicators NO_2_—PM_10_ and NO_2_—PM_2.5_ versus bronchiolitis visits by including data collected at the three centers object of the study (reported in the legend)—Model 1 and Model 2, respectively; while panels (C) and (D) include data collected only in Bologna for the same indicators—Model 3 and Model 4. Panels (E) and (F) show the association between the indicators NO_2_—EC and NO_2_—OC versus bronchiolitis visits at Bologna—Model 5 and Model 6, respectively.

All conventional air quality indicators (NO_2_, PM_10_, and PM_2.5_) demonstrate statistically significant associations with the number of admissions, with NO_2_ showing slightly higher correlation coefficients (*ρ* values). Notably, the highest correlations were observed for OC and EC, the carbonaceous components of PM, which were only measured in Bologna. To identify potential statistically significant associations between 4‐week moving average concentrations of air quality indicators and weekly hospital admissions, we developed Poisson regression models. These models considered various combinations of the available parameters using data from all three urban areas. Additionally, a series of scatter plots were created to analyze the corresponding functional relationships. Based on the results of Table 2, six models were selected for analysis. Table 3 presents the IRRs and goodness‐of‐fit values, while Figure 3 illustrates the scatter plot of predicted versus observed values.

**TABLE 2:** Correlation (Spearman's *ρ*) between the monthly mobile average of pollutants and the number of visits in the following week.

	NO_2_		PM_10_		PM_2.5_		OC		EC	
	*ρ*	*p* Value	*ρ*	*p* Value	*ρ*	*p* Value	*ρ*	*p* Value	*ρ*	*p* Value
Bologna	.615	<.001	.547	<.001	.608	<.001	.707	<.001	.638	<.001
Biella	.592	<.001	.391	<.001	.387	<.001				
Belluno	.505	<.001	.472	<.001	.465	<.001				
Overall	.447	<.001	.568	<.001	.606	<.001	.707	<.001	.638	<.001

**TABLE 3:** Model parameters and goodness‐of‐fit measures, as introduced in Section 2.4.

Parameter	IRR	95% CI		*p* Value	AIC	BIC	Log‐Likelihood	Pseudo *R* ^2^
		Lower	Upper					
Model 1 (3 centres, *n* = 2201)								
Bologna	REF.				8324	8359	−4156	.574
Biella	0.144	0.135	0.155	<.001				
Belluno	0.109	0.099	0.120	<.001				
NO_2_	1.072	1.062	1.083	<.001				
PM_10_	1.050	1.038	1.061	<.001				
NO_2_ * PM_10_ (interaction term)	0.999	0.998	0.999	<.001				
Model 2 (3 centres, *n* = 2201)								
Bologna	REF.				8292	8326	−4140	.573
Biella	0.165	0.153	0.177	<.001				
Belluno	0.109	0.100	0.120	<.001				
NO_2_	1.077	1.069	1.085	<.001				
PM_2.5_	1.066	1.055	1.077	<.001				
NO_2_ * PM_2.5_ (interaction term)	0.999	0.998	0.999	<.001				
Model 3 (Bologna, *n* = 709)								
NO_2_	1.037	1.025	1.049	<.001	4441	4459	−2217	.227
PM_10_	1.023	1.010	1.037	.001				
NO_2_ * PM_10_ (interaction term)	1.000	1.000	1.000	.995				
Model 4 (Bologna, *n* = 687)								
NO_2_	1.043	1.034	1.052	<.001	4289	4307	−2140	.233
PM_2.5_	1.034	1.023	1.046	<.001				
NO_2_ * PM_2.5_ (interaction term)	1.000	0.999	1.000	.025				
Model 5 (Bologna, *n* = 543)								
NO_2_	1.079	1.068	1.090	<.001	3434	3451	−1713	.236
EC	4.484	3.401	5.912	<.001				
NO_2_ * EC (interaction term)	0.967	0.960	0.975	<.001				
Model 6 (Bologna, *n* = 594)								
NO_2_	1.043	1.031	1.056	<.001	3657	3674	−1824	.240
OC	1.389	1.285	1.501	<.001				
NO_2_ * OC (interaction term)	0.995	0.993	0.997	<.001				

Models 1 and 2 examine the association between conventional air quality indicators (NO_2_, PM_2.5_, and PM_10_) and bronchiolitis visits, utilizing data collected from all three centers. Models 3 and 4 are similar but focus solely on data from Bologna. These models were employed for two main reasons: (a) to determine whether the relationship between air quality indicators and bronchiolitis is consistent even only at the Bologna site, which has a significantly larger dataset compared to Biella and Belluno; and (b) to establish a calibrated reference for Bologna, allowing for a more detailed exploration of the impacts of the carbonaceous components of PM, namely OC and EC, which are only available for the urban area of Bologna, primarily in Models 5 and 6.

Considering the conventional air quality indicators, the strongest associations were always found considering NO_2_ and PM_2.5_ as opposed to the combination of NO_2_ and PM_10_ (Models 1, 2, 3, and 4). This suggests a closest relation between bronchiolitis cases and the finest fraction of atmospheric PM across all the cities included in this study (Models 1 and 2) and specifically at Bologna (Models 3 and 4). Regarding the role of carbonaceous matter measured from PM_2.5_ samples, Model 6 showed the highest *R* ^2^ value (=0.45) for the relationship between NO_2_ and OC in Bologna, while Model 5 exhibited a similar performance with an *R* ^2^ value of 0.43 for the relationship between NO_2_ and EC.

The values of the IRR presented in Table 3 indicate the increase in the incidence rate, specifically the number of PED visits, that corresponds to a unit increase in the reference variable. For Models 3, 4, 5, and 6 (referred to data from Bologna), this translates to predicted increases in PED visits of 2% for a unit increase of exposure to PM_10_, 3% for PM_2.5_, 348% for EC, and 39% for OC. For NO_2_, the increase in visits falls within a range of 4% to 8% in the same models. Notably, increasing concentrations of EC, in particular, and OC significantly enhance the risk of PED visits more than the other pollutants.

## 4 DISCUSSION

This study evaluated the association between bronchiolitis admissions to the PED and air pollution in Northern Italy's urban area, a well‐known polluted hotspot in Europe. The analysis included both conventional (NO_2_, PM_2.5_, and PM_10_) and nonconventional air quality indicators, specifically OC and EC. These components of the carbonaceous fraction of atmospheric PM are associated with specific pollution sources such as vehicular traffic and biomass burning within the study area.

We found a significant correlation between bronchiolitis cases and exposure to conventional pollutants across three urban areas. These results are in line with the literature, reporting peaks in bronchiolitis cases coinciding with higher exposure to airborne pollutants levels, although overwhelming evidence is yet least and, in some cases, conflicting (Table S4).

The main limit of methodological approaches is the difficulty in assessing exposure to PM, considered the main driver of health outcomes related to air pollution. This is due to the absence of standardized methodologies to characterize the properties of particle populations that may be more relevant for health‐related issues. Indeed, the international air quality standards primarily rely on PM mass concentration that integrates all particles within a certain bulk chemical composition and size range, providing little information about their specific features connected to actual biological outcomes.

The WHO recognizes that not all components of PM are equally significant for health effects.^9^ The intricate mixture of PM_2.5_ and PM_10_ encompasses liquid and solid particles suspended in the atmosphere, varying in size, chemical composition, origin, shape, and mixing state.^8^ These particles undergo complex atmospheric aging processes and display varying levels of toxicity.^22^, ^23^, ^24^, ^25^ Current PM mass metrics fail to capture this complexity, and there is a recognized need for novel metrics in research and legislation^26^; nevertheless, no definitive alternative has been identified. For instance, the mass concentration of certain components of PM_1_ or PM_2.5_, such as EC, redox‐active transition metals, and secondary organic aerosol (SOA), the number concentration in certain size‐ranges, such as below 100 nm, as well as their potential to generate reactive oxygen species (ROS) and produce oxidative stress have been proposed.^8^

To align with the latest recommendations, for the site of Bologna, the carbonaceous fractions of PM, OC, and EC are utilized as supplementary, nonconventional air quality indicators. These components provide valuable insights into the physicochemical properties of PM composition, enabling the tracing of contributions from specific sources and formation mechanisms relevant to the environmental context of the study.

The results from the Poisson linear model, including NO_2_ and PM_2.5_ and, as a second step, NO_2_ with EC, and NO_2_ with OC, indicated that the two latter combinations performed better, even though slightly, in predicting weekly bronchiolitis admissions to the PED in Bologna. This suggests that exposure to the carbonaceous components of PM may be relevant in determining the worsening of bronchiolitis symptoms. In contrast, the total mass of PM (PM_2.5_ or PM_10_), which includes other chemical components like ammonium nitrate that are less significant in terms of health effects, may be less relevant for this outcome.

According to the model parameters and goodness‐of‐fit measures (Table 3), EC is the variable that most significantly increases the IRR, with each additional microgram per cubic meter in the atmosphere resulting in more than a tripling of hospitalizations. OC also raises the IRR considerably, but to a lesser extent, contributing to a 40% increase in hospital admissions for each additional microgram. This different impact may be related to their different concentration ranges observed in our datasets: an increase of one unit of OC concentration results in a 15%–30% rise, whereas adding 1 μg/m^3^ of EC leads to a substantial increase of 55%–120% in its concentrations. We may also speculate that EC is typically associated with smaller, more toxic particles compared to OC, which may enhance its ability to penetrate the respiratory system and lead to adverse health effects.^23^, ^27^ Recent literature supports this, indicating that the highest pro‐inflammatory and oxidative responses occur when urban aerosols are dominated by ultrafine, EC‐enriched particles from fossil fuel combustion, even at low overall PM mass concentrations.^23^

Our findings demonstrate that the carbonaceous fraction is a relevant component of urban PM exacerbating bronchiolitis and that it can be considered a more effective proxy compared to total PM mass (PM_10_ and PM_2.5_). Our dataset highlights important sources of wintertime pollution, particularly combustion processes,^12^, ^28^ while it does not allow us to determine whether fossil fuel combustion—primarily related to traffic—or biomass burning for domestic heating has a greater influence on the negative health outcomes examined.

Stronger correlations were observed with a 4‐week compared to a 1‐week exposure period, indicating that medium‐term exposure has a more pronounced impact on the risk of hospitalization for bronchiolitis than short‐term exposure. The duration of exposure to air pollution is a controversial point (Table S4); our data emphasize the importance of longer exposure. This could be explained by the development of an inflamed microenvironment in the child's lung that potentially promotes bronchiolitis in infants that are prone to oxidative stress.

Based on these results, we hypothesize that developing strategies to reduce the emissions of air pollution may contribute to reaching a potential dual purpose: to reduce the burden of bronchiolitis during seasonal peaks, but also prospectively they may have an impact on the risk of developing asthma, which is a well‐known consequence in a non‐negligible proportion of patients with bronchiolitis, especially if caused by RSV or RV.^29^

Early childhood exposures can affect the airways and immune system developmental trajectories, with reduced lung function and asthma pathogenesis. From a pathophysiological perspective, air quality impairment has been associated with a decrease in CC16, a biomarker whose decrease has been associated with oxidative stress and reduced lung function. Airborne pollutants may influence asthma development through altered immune development, increased IgE‐mediated allergic sensitization, Th17‐associated responses, and oxidative stress, causing inflammation.^30^

These long‐term consequences are also confirmed by a recently published multicohort study showing that exposure to high air pollution levels in the first 3 years of life is associated with an increased risk of developing asthma.^31^

This study presents limitations: the experimental design did not allow for a detailed characterization of specific air pollution features that would better define airborne particles based on multiple physicochemical properties. This hampers the identification of specific PM characteristics as the primary drivers of health outcomes. Future epidemiological studies should adopt multi‐criteria metrics to better understand bronchiolitis development. Additionally, the separate analysis of healthcare utilization and environmental data introduces biases due to the ecological design, which relies on population‐level data and limits adjustment for individual exposures and risk factors (e.g., indoor air pollution, comorbidities, and siblings). Finally, considering the differences in terms of the number of patients enrolled by the Bologna center compared to Belluno and Biella, a future study may take advantage of the inclusion of larger centers to better describe the Po Valley situation.

## 5 CONCLUSIONS

This study, conducted in a well‐known polluted environment in Northern Italy, indicates that medium‐term exposure to elevated air pollution increases the risk of acute bronchiolitis.

Our results showed the associations between bronchiolitis admissions and the exposure to the carbonaceous fraction of fine PM_2.5_, representing specific anthropogenic combustion sources in the studied cohort. This finding indicates that the carbonaceous fraction may serve as a more accurate proxy for the associations between air pollution and bronchiolitis than the classical mass concentration (PM_10_, PM_2.5_).

Children are particularly vulnerable to air pollutants. Therefore, their exposure should be minimized in areas most impacted by anthropogenic pollution sources, and mitigation policies should be implemented. Additional research is needed to identify the most effective air quality metrics and the causal mechanisms linking bronchiolitis to infant exposure to specific sources of air pollution. This clarification could influence pollutant abatement strategies, allowing a focus on the most harmful sources for cost reductions and improved effectiveness.

## AUTHOR CONTRIBUTIONS

**D. Zama:** Conceptualization; investigation; writing – original draft; project administration; visualization; methodology; supervision; writing – review and editing; data curation. **A. Paccapelo:** Methodology; software; data curation; formal analysis; validation; visualization; writing – review and editing. **L. Betti:** Writing – original draft; writing – review and editing; investigation. **E. Manieri:** Investigation; writing – original draft; writing – review and editing. **M. Paglione:** Writing – original draft; writing – review and editing; data curation; project administration; methodology. **M. Rinaldi:** Data curation; writing – original draft; writing – review and editing; project administration; methodology. **A. Dondi:** Conceptualization; investigation; writing – original draft; writing – review and editing; project administration; methodology; supervision; visualization; data curation. **E. Battelli:** Project administration; methodology; data curation. **C. Biagi:** Conceptualization; project administration; writing – review and editing; methodology; data curation; supervision. **C. Marchegiani Rizzolli:** Writing – review and editing; investigation; conceptualization; data curation. **P. Manzoni:** Conceptualization; methodology; writing – review and editing; supervision. **G. Piglia:** Data curation; conceptualization; writing – review and editing; investigation. **G. Nicolini:** Conceptualization; methodology; writing – review and editing; supervision. **M. Lanari:** Conceptualization; methodology; writing – review and editing; supervision; project administration. **C. Carbone:** Conceptualization; investigation; writing – original draft; writing – review and editing; project administration; data curation; methodology.

## FUNDING INFORMATION

This research received no external funding.

## CONFLICT OF INTEREST STATEMENT

The authors declare no conflict of interest.

### PEER REVIEW

The peer review history for this article is available at https://www.webofscience.com/api/gateway/wos/peer‐review/10.1111/pai.70077.

## ETHICS STATEMENT

The study was conducted in accordance with the Declaration of Helsinki, and it was approved by the Institutional Reviewer Board of Area Vasta Emilia Centro, AVEC (study Number 1062/2020/Oss/AOUBo and subsequent amendment in 2022). A written consent was obtained from the parents or guardians of the children involved in this study.

## Supporting information


Appendix S1.
