# Supplementary material for: The influence of air pollutants on the risk of emergency department presentations of infants with bronchiolitis in an European air quality hotspot
Source: Pediatr Allergy Immunol. 2025 Apr 2;36(4):e70077. doi: 10.1111/pai.70077 (PMC11963223; doi:10.1111/pai.70077)
Supplement: Supplementary file 1 — Appendix S1. [file PAI-36-e70077-s001.docx]

**Supplementary Material:**

Table S1 – Clinical data collection: for each patient we considered the following data.

| **Category of variables** | **Variables considered** |
| --- | --- |
| Demographic Data | - Sex - Age in months - Weight - Ethnicity |
| Known risk factors for the development of bronchiolitis | - Previous episodes of apnea - Wheezing - Chronic pulmonary disease - Congenital heart disease - Immunodeficiency - Severe neurological or muscle disease - Prematurity |
| Type of feeding | - Breastfeeding - Mixed feeding - Formula feeding |
| Vital signs at PED arrival | - Heart rate - Respiratory rate - Body temperature - Oxygen saturation |
| Discharge modality | - Home - SSOU - Admission to the pediatric ward |
| Complications | - Pneumonia - Sepsis - Pneumothorax - Pleural effusion |

*PED: Pediatric Emergency Department; SSOU: short‐stay observation unit.*

Table S2 – Correlation (Spearman’s ρ) between monthly pollutants mobile averages and daily visits

| **Centre** | **NO_2_** | | **PM_10_** | | **PM_2.5_** | | **OC** | | **EC** | | **BC** | |
| --- | --- | --- | --- | --- | --- | --- | --- | --- | --- | --- | --- | --- |
|  | ρ | p-value | ρ | p-value | ρ | p-value | ρ | p-value | ρ | p-value | ρ | p-value |
| **Bologna** | 0.390 | <0.001 | 0.349 | <0.001 | 0.383 | <0.001 | 0.439 | <0.001 | 0.406 | <0.001 | 0.431 | <0.001 |
| **Biella** | 0.284 | <0.001 | 0.185 | <0.001 | 0.183 | <0.001 |  | <0.001 |  | <0.001 |  | <0.001 |
| **Belluno** | 0.189 | <0.001 | 0.172 | <0.001 | 0.166 | <0.001 |  | <0.001 |  | <0.001 |  | <0.001 |
| **Overall** | 0.291 | <0.001 | 0.396 | <0.001 | 0.440 | <0.001 | 0.439 | <0.001 | 0.406 | <0.001 | 0.431 | <0.001 |

Table S3 – Correlation (Spearman’s ρ) between weekly pollutants mobile averages and visits

| **Centre** | **NO_2_** | | **PM_10_** | | **PM_2.5_** | | **OC** | | **EC** | | **BC** | |
| --- | --- | --- | --- | --- | --- | --- | --- | --- | --- | --- | --- | --- |
|  | ρ | p-value | ρ | p-value | ρ | p-value | ρ | p-value | ρ | p-value | ρ | p-value |
| **Bologna weekly visits** | 0.523 | <0.001 | 0.403 | <0.001 | 0.497 | <0.001 | 0.571 | <0.001 | 0.538 | <0.001 | 0.603 | <0.001 |
| **Bologna daily visits** | 0.334 | <0.001 | 0.284 | <0.001 | 0.340 | <0.001 | 0.377 | <0.001 | 0.373 | <0.001 | 0.398 | <0.001 |

Table S4 - Main studies on the relationship between air pollutants and bronchiolitis.

| Study | Years conducted | Country | Time lag | Pollutants | Significant findings |
| --- | --- | --- | --- | --- | --- |
| Karr et al.  (2004)^1^ | 1995–2000 | USA | 1-2 and 3-5 days | PM_2.5_, NO_2_, CO | No effects of PM_2.5_, NO_2_, CO on bronchiolitis |
| Karr et al.  (2006) ^2^ | 1995–2000 | USA | 1–2 and 3–5 days for PM_2.5_, 1 and  4 days for NO_2_, CO | PM_2.5_, NO_2_, CO | No association between PM_2.5_, NO_2_ and CO and bronchiolitis |
| Karr et al. (2007) ^3^ | 1995–2000 | USA | Chronic (lifetime) and sub-chronic (mean of previous 30 days) | PM_10_, NO_2_, CO, O_3_ | Association between sub-chronic and chronic exposure to PM_2.5_ and increased risk of hospitalization for bronchiolitis |
| Ségala et al. (2008) ^4^ | 1997–2001 | France | 0-1 days, 0-4 days | PM_10_,  NO_2_, SO_2_ | Association between PM_10_,  NO_2_, SO_2_ and visits and hospitalization for bronchiolitis |
| Karr et al. (2009a)^5^ | 1999–2002 | Canada | Lifetime and first month | PM_2.5_, PM_10_, BC  NO_2_, NO, SO_2_,  CO, O_3_ | Association between lifetime exposure to NO_2_, NO, SO_2_,  CO and increased risk of bronchiolitis. No association between exposure to PM_2.5_, PM_10_ e BC. Negative correlation between bronchiolitis and O_3_ |
| Karr et al. (2009b)^6^ | 1997–2003 | USA | Lifetime and mean of previous 30, 60 days, mean of 7 days (only for PM_2.5_) | PM_2.5_, NO_2_ | No association between PM_2.5_ and NO_2_ and bronchiolitis |
| Sheffield et al. (2011) ^7^ | 1999 - 2007 | USA | Lifetime | PM_2.5_, PM_10_,  NO_2_, SO_2_,  CO, O_3_ | Association between chronic exposure to PM_2.5_ and hospitalization for bronchiolitis |
| Vandini et al. (2013)^8^ | 2007 - 2010 | Italy | Weekly mean of the previous week | PM_2.5_, PM_10_ | Correlation between RSV bronchiolitis and mean PM_10_ concentration of the previous week. No correlation with PM_2.5_ |
| Darrow et al. (2014) ^9^ | 1993 - 2004 | USA | 0-3 days | PM_2._5, PM10,  NO_2_, O_3_ | No association between PM_2.5_, PM_10_, NO_2_, O_3_ and bronchiolis |
| Evangelisti et al. (2015) ^10^ | 2004 -2014 | Italy | Not defined | PM_2.5_, PM_10_,  NO_2_, NO, SO_2_,  CO, O_3_, C_6_H_6_ | Correlation between RSV bronchiolitis and PM_2.5_, PM_10_,  NO_2_, NO, C_6_H_6_ and negative correlation with O_3_ |
| Pablo-Romero et al. (2015)^11^ | 2007 - 2011 | Spain | Not defined | PM_2.5_ | Association between PM_2.5_ increase and bronchiolitis |
| Mohammed et al. (2016)^12^ | 2011 - 2012 | UK | Various short-term time lag | NO | Association between RSV bronchiolitis and NO exposure |
| Yitshak-sade et al. (2017) ^13^ | 2003 - 2013 | Israel | 0–1, 0–4, e  0–7 days | PM_2.5_, PM_10_, SO_2_, NO_2_ | Association between high PM_2.5_, PM_10_ and NO_2_ level and bronchiolitis |
| Girguis  et al.  (2017) ^14^ | 2001–2009 | USA | Lifetime | PM_2.5_ | No association between chronic exposure to PM_2.5_ and bronchiolitis |
| Abdul  Rahman  et al.  (2017) ^15^ | 2006–2010 | Malesia | Lifetime | PM_10_, NO_2_, CO, O_3_ | Association between PM_10_ and bronchiolitis |
| Nenna et al. (2017) ^16^ | 2004 -2014 | Italy | 1 week | PM_2.5_, PM_10_,  NO_2_, SO_2_,  CO, O_3_, C_6_H_6_ | Correlation between RSV bronchiolitis and PM_2.5_, PM_10_,  NO_2_, SO_2_, CO, C_6_H_6_; negative correlation with O_3_; association between C_6_H_6_ and hospitalization for bronchiolitis |
| Carugno et al. (2018) ^17^ | 2012–2013 | Italy | Daily mean 0-30 days, mean 0-30 days, previous 4 weeks | PM_10_ | Association between short- and medium-term exposure to PM_10_ and risk of hospitalization for RSV bronchiolitis |
| Girguis  et al.  (2018) ^18^ | 2001 –2008 | USA | 1, 4, 7 days | PM_2.5_ | Increased risk of bronchiolitis for PM_2.5_ exposure for lag 1 and 4 day, but not for 7 days |
| Horne et al. (2018) ^19^ | 1999 - 2016 | USA | 0–6, 7–13, 14–20 and 21–27 previous days | PM_2.5_ | Association between short-term exposure to PM_2.5_ and bronchiolitis |
| Kennedy et al. (2018) ^20^ | 2000 - 2010 | USA | First year of life | PM_2.5_, NO_x_, CO | Modest positive association between bronchiolitis and traffic emissions |
| Martín Martín et al. (2018) ^21^ | 2013 - 2015 | Spain | Not defined | NO_2_, SO_2_, CO, NO_x_, C_6_H_6_, PM_2.5_, PM_10_, O_3_, | Association between NO_2_ and bronchiolitis |
| Terrazas et al. (2019) ^22^ | 2001 - 2014 | Chile | Not defined | PM_2.5_ | Direct correlation between PM_2.5_ and hospitalization for bronchiolitis |
| Matus et al. (2019) ^23^ | 2001 - 2005 | Chile | 2 days | PM_2.5_, PM_10_, O_3_, | Association between short-term exposure and hospitalization for bronchiolitis |
| Cheng et al. (2020) ^24^ | 2013 - 2015 | Australia | 1 day | PM_2.5_, PM_10_, NO_2_, O_3_ | Association between short-term exposure and bronchiolitis |
| Kim et al. (2020) ^25^ | 2013 - 2015 | Korea | Short-term | PM_2.5_ | Association between increased PM_2.5_ levels and bronchiolitis |
| Esteban et al. (2020) ^26^ | 2011 - 2016 | Spain | 10 days | PM_2.5_, PM_10_, NO_2_, SO_2_ | No association between pollutants and hospitalization for bronchiolitis |
| Ortega-García et al. (2020) ^27^ | 2015 | Spain | Not defined | PM_10_, NO_2_, O_3_, SO_2_ | Air pollution increases hospitalization for bronchiolitis |
| Cordova et al. (2020) ^28^ | 2011 - 2015 | Peru | Varius time lag | PM_2.5_ | Association between PM_2.5_ and bronchiolitis |
| Luong et al. (2020) ^29^ | 2016 - 2017 | Vietnam | 0-3 days | PM_2.5_ | Association between PM_2.5_ and bronchiolitis |
| Leung et al. (2021) ^30^ | 2008 - 2017 | China | Varius time lag | SO_2_, O_3_, NO_2_, PM_10_ | Correlation between hospitalization for bronchiolitis and NO_2_ and PM_10_ exposure |
| Liang et al. (2021) ^31^ | 2013 - 2019 | China | 0-5 days | PM_2.5_, PM_10_, coarse particulate matter, NO_2_, SO_2_,  O_3_ | Association between PM_2.5_, PM_10_, coarse particulate matter and bronchiolitis |
| Wrotek et al. (2021) ^32^ | 2010 - 2019 | Poland | Not defined | PM_2.5_, PM_10_,  NO_2_ | Association between PM_2.5_, PM_10_, NO_2_ and hospitalization for bronchiolitis |
| Milani et al. (2022) ^33^ | 2019 - 2020 | Italy | Daily mean of the previous 29 days | PM_2.5_, PM_10_ | Direct association between PM_2.5_ and PM_10_ levels and bronchiolitis severity |
| Chen et al. (2022) ^34^ | 2006 - 2016 | Taiwan | Varius time lag | PM_2.5_ | Association between PM_2.5_ and bronchiolitis |
| Gallo et al. (2022) ^35^ | 2007 - 2018 | Italy | 0-14 days | PM_2.5_, PM_10_,  NO_2_ | Association between PM_2.5_, PM_10_,  NO_2_ and visits and hospitalization for bronchiolitis |
| Xiao et al. (2022) ^36^ | 2013 - 2019 | China | 0-5 days | PM_2.5_, PM_10_,  NO_2_, SO_2_,  O_3_ | Association between PM_2.5_ and bronchiolitis |
| Liang et al. (2022) ^37^ | 2014 - 2016 | China | 0-5 days | PM_2.5_, PM_10_, coarse particulate matter, NO_2_, SO_2_,  O_3_ | Association between PM_2.5_, PM_10_, coarse particulate matter and bronchiolitis |
| Dondi et al. (2023)^38^ | 2011 - 2020 | Italy | Mean of the previous week and 4 weeks | PM_2.5_, PM_10_,  NO_2_,  C_6_H_6_ | PM_2,5_ exposure in the previous 4 weeks is the main factor increasing significantly the risk of hospitalization for bronchiolitis |
| Wang et al. (2023) ^39^ | 2019 - 2022 | China | Various time lag | PM_2.5_, PM_10_, SO_2_, NO_2_, CO, O_3_ | Association between PM_2.5_, PM_10_, SO_2_, NO_2_, CO and bronchiolitis |
| Zhang et al. (2023) ^40^ | 2014 - 2020 | China | Short-term | PM_2.5_, PM_10_, SO_2_, NO_2_ | Association between PM_2.5_, PM_10_, SO_2_, NO_2_ and bronchiolitis |
| Villamil-Osorio et al. (2023) ^41^ | 2019 - 2021 | Colombia | Not defined | NO_2_, CO PM_2.5_, PM_10_, SO_2_, O_3_ | Negative correlation between NO_2_ and CO and bronchiolitis |
| Lei et al. (2023) ^42^ | 2013 - 2020 | China | 0-4 days | NO_2_, CO PM_2.5_, PM_10_, SO_2_, O_3_ | Association between PM_2.5_ and PM_10_ and bronchiolitis |
| Brusselen et al. (2024) ^43^ | 2020 - 2021 | Belgium | Short-term (1-5 days) and medium-term (31 days) | PM_2.5_, PM_10_,  BC, NO_2_ | Association between medium-term exposure to PM_10_ and NO_2_ and severe bronchiolitis |


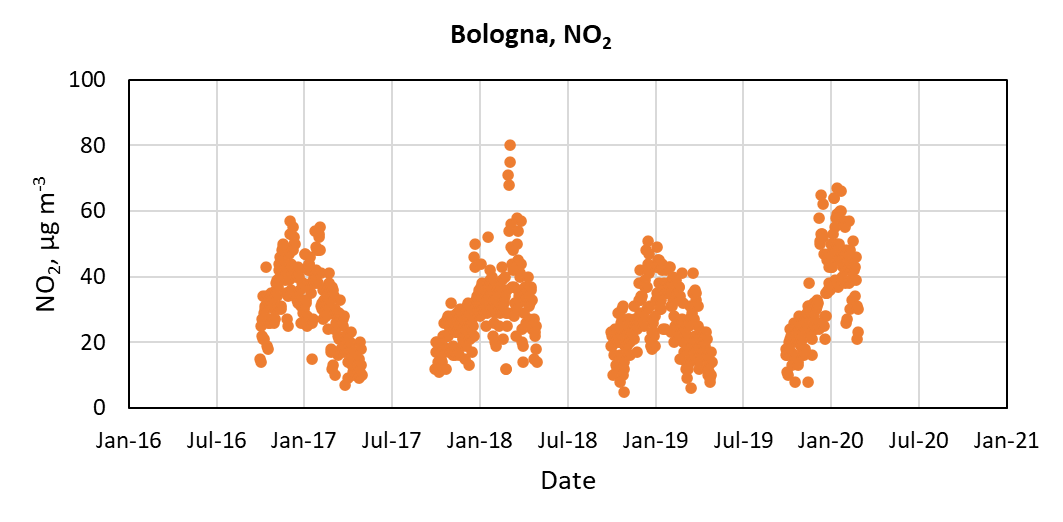


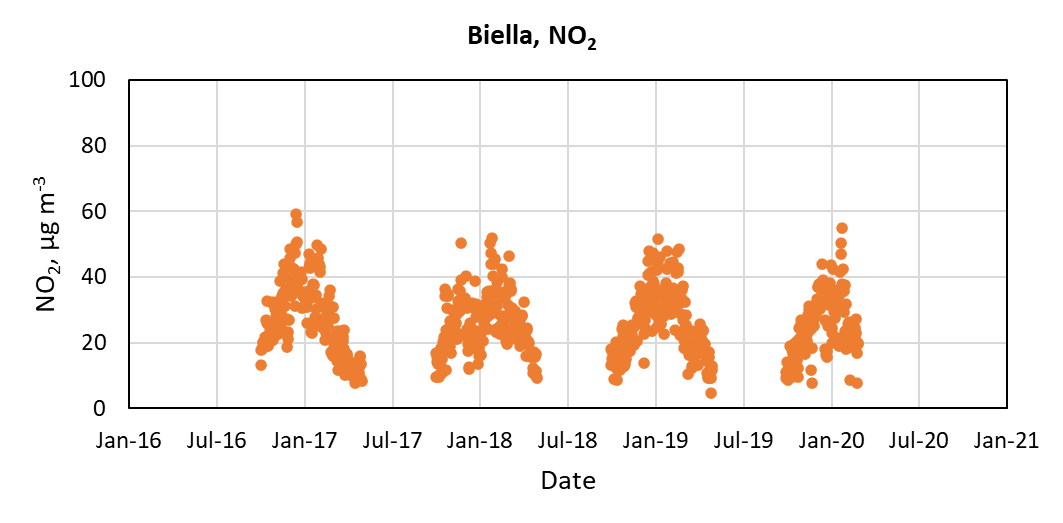


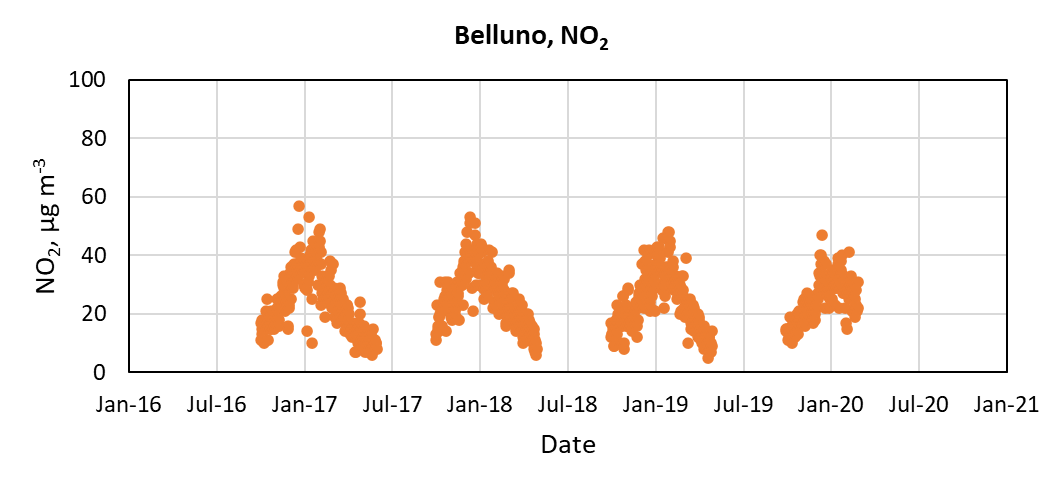


**Figure S1.** Daily concentrations of NO_2_ in the cities of Bologna, Biella and Belluno. Only days within the epidemiological season (October-March) have been considered.


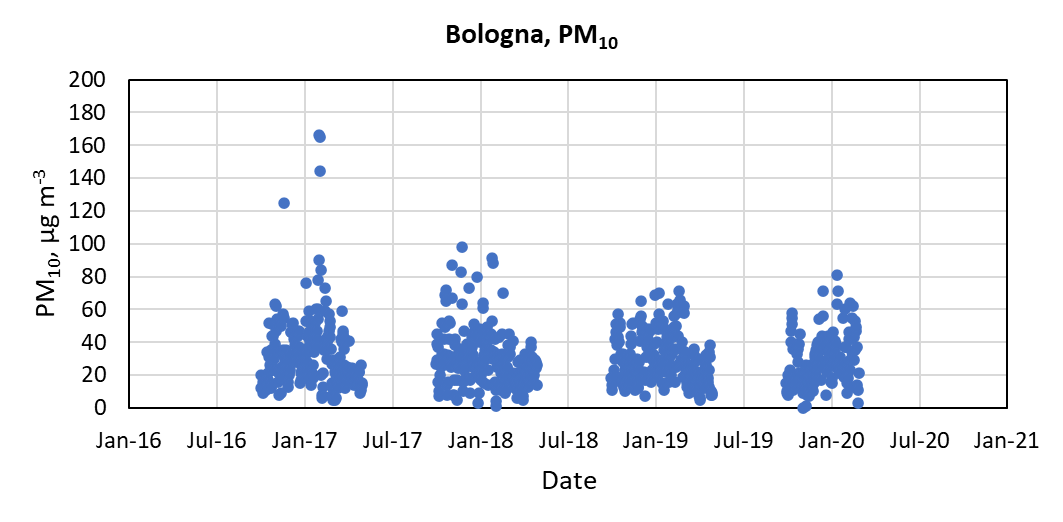


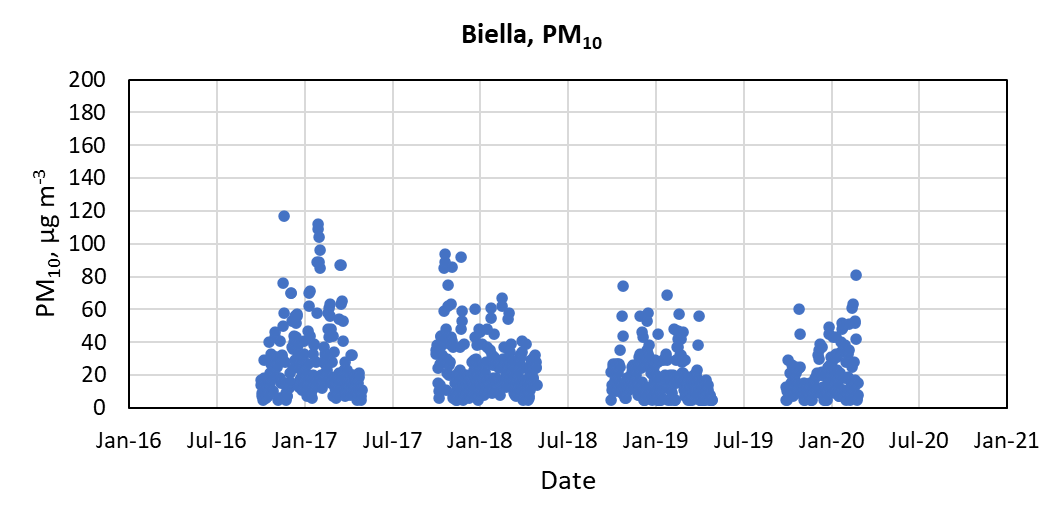


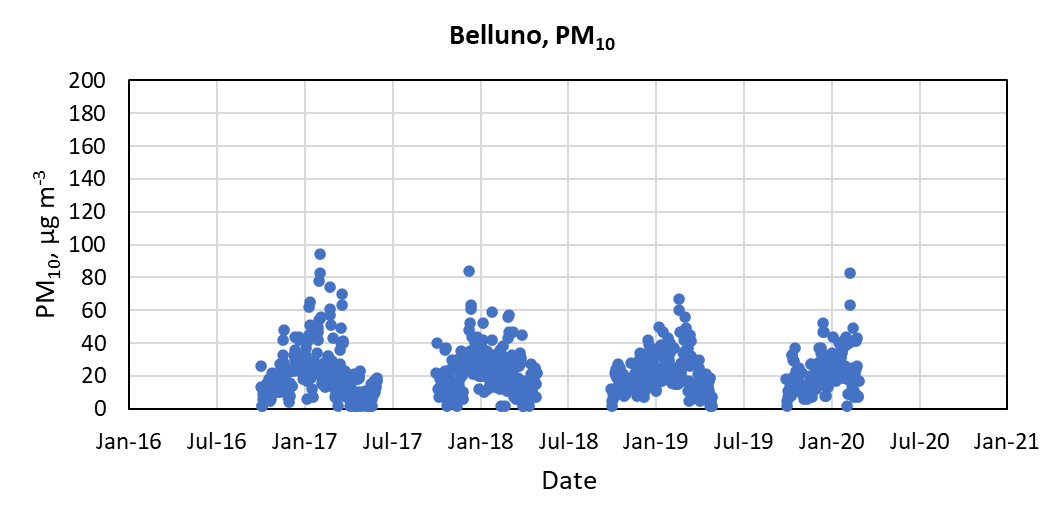


**Figure S2.** Daily concentrations of PM_10_ in the cities of Bologna, Biella and Belluno. Only days within the epidemiological season (October-March) have been considered.


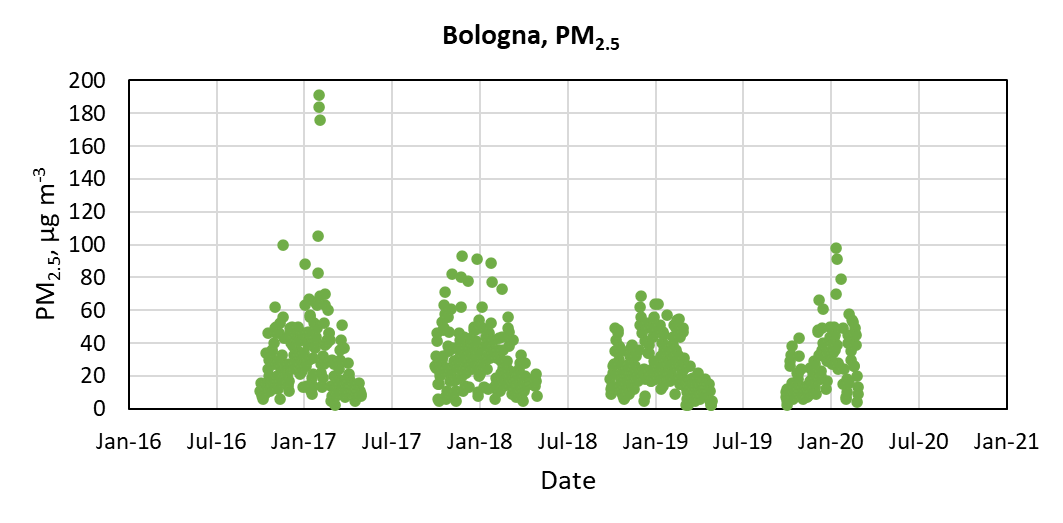


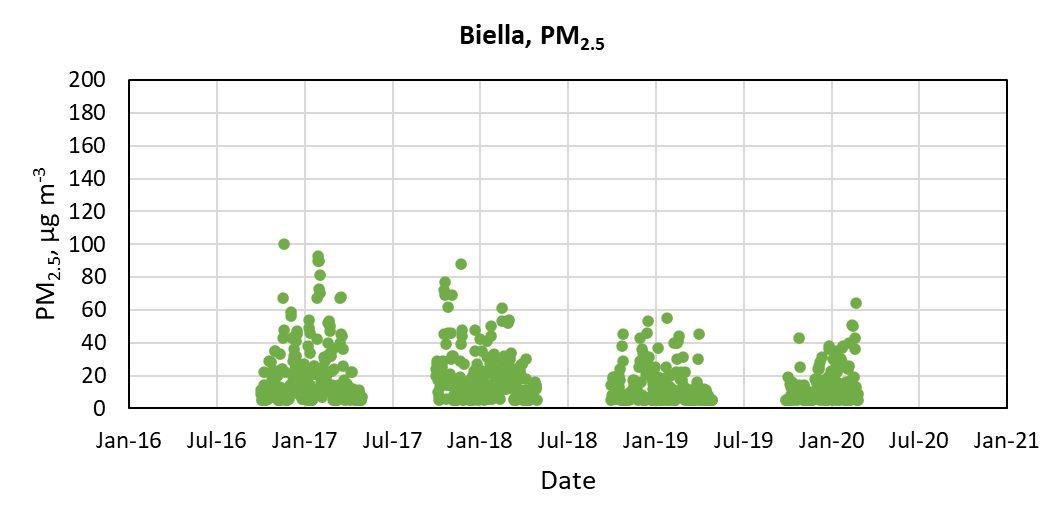


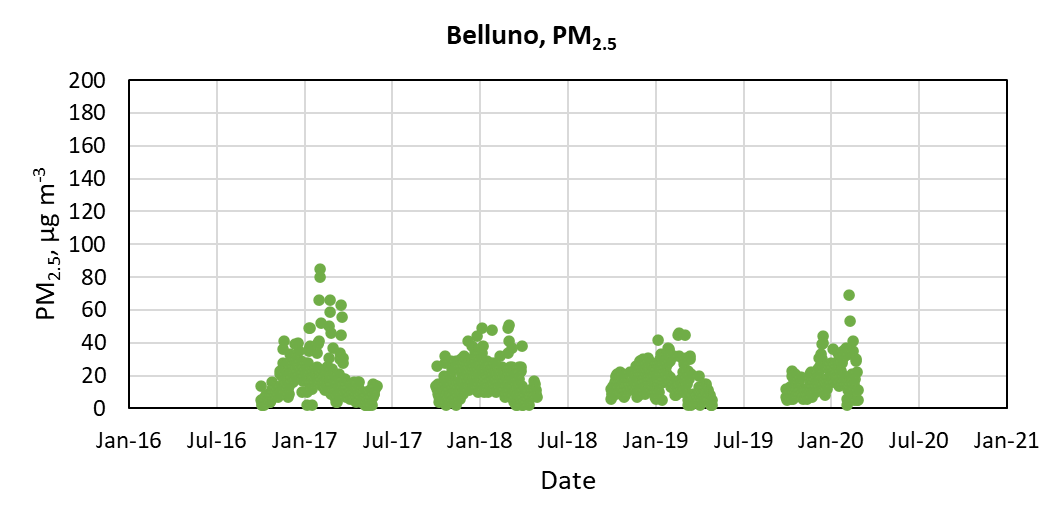


**Figure S3.** Daily concentrations of PM_2.5_ in the cities of Bologna, Biella and Belluno. Only days within the epidemiological season (October-March) have been considered.


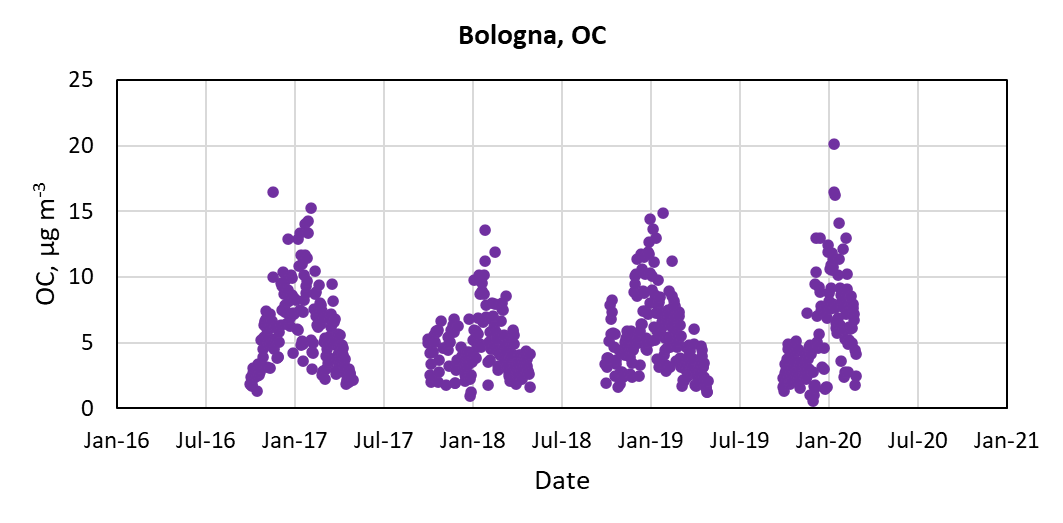


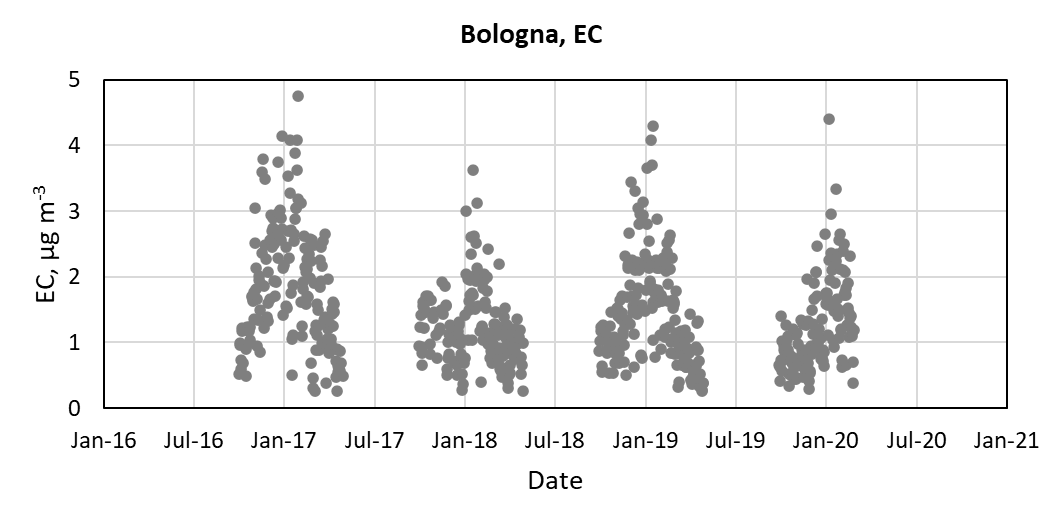


**Figure S4.** Daily concentrations of OC and EC in the city of Bologna. Only days within the epidemiological season (October-March) have been considered.

**REFERENCES**

1. Karr C, Kaufman J, Lumley T, et al. EFFECT OF AMBIENT AIR POLLUTION ON INFANT BRONCHIOLITIS. *Epidemiology*. 2004;15(4):S31.

2. Karr C, Lumley T, Shepherd K, et al. A case-crossover study of wintertime ambient air pollution and infant bronchiolitis. *Environ Health Perspect*. 2006;114(2):277-281. doi:10.1289/ehp.8313

3. Karr C, Lumley T, Schreuder A, et al. Effects of subchronic and chronic exposure to ambient air pollutants on infant bronchiolitis. *Am J Epidemiol*. 2007;165(5):553-560. doi:10.1093/aje/kwk032

4. Ségala C, Poizeau D, Mesbah M, Willems S, Maidenberg M. Winter air pollution and infant bronchiolitis in Paris. *Environmental Research*. 2008;106(1):96-100. doi:10.1016/j.envres.2007.05.003

5. Karr CJ, Demers PA, Koehoorn MW, Lencar CC, Tamburic L, Brauer M. Influence of ambient air pollutant sources on clinical encounters for infant bronchiolitis. *Am J Respir Crit Care Med*. 2009;180(10):995-1001. doi:10.1164/rccm.200901-0117OC

6. Karr CJ, Rudra CB, Miller KA, et al. Infant exposure to fine particulate matter and traffic and risk of hospitalization for RSV bronchiolitis in a region with lower ambient air pollution. *Environ Res*. 2009;109(3):321-327. doi:10.1016/j.envres.2008.11.006

7. Sheffield P, Roy A, Wong K, Trasande L. Fine particulate matter pollution linked to respiratory illness in infants and increased hospital costs. *Health Aff (Millwood)*. 2011;30(5):871-878. doi:10.1377/hlthaff.2010.1279

8. Vandini S, Corvaglia L, Alessandroni R, et al. Respiratory syncytial virus infection in infants and correlation with meteorological factors and air pollutants. *Ital J Pediatr*. 2013;39(1):1. doi:10.1186/1824-7288-39-1

9. Darrow LA, Klein M, Flanders WD, Mulholland JA, Tolbert PE, Strickland MJ. Air pollution and acute respiratory infections among children 0-4 years of age: an 18-year time-series study. *Am J Epidemiol*. 2014;180(10):968-977. doi:10.1093/aje/kwu234

10. Evangelisti M, Cangiano G, Nenna R, et al. Air pollution and bronchiolitis from 2004 to 2014 in Rome. *European Respiratory Journal*. 2015;46(suppl 59). doi:10.1183/13993003.congress-2015.PA4505

11. de P Pablo-Romero M, Román R, Limón JMG, Praena-Crespo M. Effects of fine particles on children’s hospital admissions for respiratory health in Seville, Spain. *J Air Waste Manag Assoc*. 2015;65(4):436-444. doi:10.1080/10962247.2014.1001499

12. Mohammed NI, Everard ML, Ayres JG, Barker NJ, Litchfield IJ. A Preliminary Assessment of the Role of Ambient Nitric Oxide Exposure in Hospitalization with Respiratory Syncytial Virus Bronchiolitis. *Int J Environ Res Public Health*. 2016;13(6):578. doi:10.3390/ijerph13060578

13. Yitshak-Sade M, Yudovitch D, Novack V, Tal A, Kloog I, Goldbart A. Air Pollution and Hospitalization for Bronchiolitis among Young Children. *Ann Am Thorac Soc*. 2017;14(12):1796-1802. doi:10.1513/AnnalsATS.201703-191OC

14. Girguis MS, Strickland MJ, Hu X, et al. Chronic PM2.5 exposure and risk of infant bronchiolitis and otitis media clinical encounters. *Int J Hyg Environ Health*. 2017;220(6):1055-1063. doi:10.1016/j.ijheh.2017.06.007

15. Abdul Rahman SR, Ismail SNS, Sahani M et al. A case crossover analysis of primary air pollutants association on acute respiratory infection (ARI) among children in urban region of Klang valley, Malaysia. *Ann Trop Med Public Heal*. Published online 2017. doi:10(1):44-55. doi:10.4103/ATMPH.ATMPH_75_17

16. Nenna R, Evangelisti M, Frassanito A, et al. Respiratory syncytial virus bronchiolitis, weather conditions and air pollution in an Italian urban area: An observational study. *Environ Res*. 2017;158:188-193. doi:10.1016/j.envres.2017.06.014

17. Carugno M, Dentali F, Mathieu G, et al. PM10 exposure is associated with increased hospitalizations for respiratory syncytial virus bronchiolitis among infants in Lombardy, Italy. *Environmental Research*. 2018;166:452-457. doi:10.1016/j.envres.2018.06.016

18. Girguis MS, Strickland MJ, Hu X, et al. Exposure to acute air pollution and risk of bronchiolitis and otitis media for preterm and term infants. *Journal of Exposure Science and Environmental Epidemiology*. 2018;28(4). doi:https://doi-org.ezproxy.unibo.it/10.1038/s41370-017-0006-9

19. Horne BD, Joy EA, Hofmann MG, et al. Short-Term Elevation of Fine Particulate Matter Air Pollution and Acute Lower Respiratory Infection. *Am J Respir Crit Care Med*. 2018;198(6):759-766. doi:10.1164/rccm.201709-1883OC

20. Kennedy CM, Pennington AF, Darrow LA, et al. Associations of mobile source air pollution during the first year of life with childhood pneumonia, bronchiolitis, and otitis media. *Environ Epidemiol*. 2018;2(1):e007. doi:10.1097/EE9.0000000000000007

21. Martín Martín R, Sánchez Bayle M. [Impact of air pollution in paediatric consultations in Primary Health Care: Ecological study]. *An Pediatr (Engl Ed)*. 2018;89(2):80-85. doi:10.1016/j.anpedi.2017.06.013

22. Terrazas C, Castro-Rodriguez JA, Camargo Jr CA, Borzutzky A. Solar radiation, air pollution, and bronchiolitis hospitalizations in Chile: An ecological study. *Pediatric Pulmonology*. 2019;54(9):1466-1473. doi:10.1002/ppul.24421

23. Matus C P, Oyarzún G M. [Impact of Particulate Matter (PM 2,5 ) and children’s hospitalizations for respiratory diseases. A case cross-over study]. *Rev Chil Pediatr*. 2019;90(2):166-174. doi:10.32641/rchped.v90i2.750

24. Cheng J, Su H, Xu Z. Intraday effects of outdoor air pollution on acute upper and lower respiratory infections in Australian children. *Environ Pollut*. 2021;268(Pt A):115698. doi:10.1016/j.envpol.2020.115698

25. Kim KN, Kim S, Lim YH, Song IG, Hong YC. Effects of short-term fine particulate matter exposure on acute respiratory infection in children. *Int J Hyg Environ Health*. 2020;229:113571. doi:10.1016/j.ijheh.2020.113571

26. Esteban ME, Bote-González M, Alejandre C, Balaguer M, Jordan I. [Influence of meteorological factors and air pollutants on severe bronchiolitis cases in the metropolitan area of Barcelona: A pilot study]. *An Pediatr (Engl Ed)*. 2020;92(4):229-231. doi:10.1016/j.anpedi.2019.01.026

27. Ortega-García JA, Martínez-Hernández I, Boldo E, et al. [Urban air pollution and hospital admissions for asthma and acute respiratory disease in Murcia city (Spain)]. *An Pediatr (Engl Ed)*. 2020;93(2):95-102. doi:10.1016/j.anpedi.2020.01.012

28. Davila Cordova JE, Tapia Aguirre V, Vasquez Apestegui V, et al. Association of PM2.5 concentration with health center outpatient visits for respiratory diseases of children under 5 years old in Lima, Peru. *Environ Health*. 2020;19(1):7. doi:10.1186/s12940-020-0564-5

29. Luong LTM, Dang TN, Thanh Huong NT, et al. Particulate air pollution in Ho Chi Minh city and risk of hospital admission for acute lower respiratory infection (ALRI) among young children. *Environ Pollut*. 2020;257:113424. doi:10.1016/j.envpol.2019.113424

30. Leung SY, Lau SYF, Kwok KL, Mohammad KN, Chan PKS, Chong KC. Short-term association among meteorological variation, outdoor air pollution and acute bronchiolitis in children in a subtropical setting. *Thorax*. 2021;76(4):360-369. doi:10.1136/thoraxjnl-2020-215488

31. Liang Z, Meng Q, Yang Q, Chen N, You C. Size-Specific Particulate Matter Associated With Acute Lower Respiratory Infection Outpatient Visits in Children: A Counterfactual Analysis in Guangzhou, China. *Front Public Health*. 2021;9:789542. doi:10.3389/fpubh.2021.789542

32. Wrotek A, Badyda A, Czechowski PO, Owczarek T, Dąbrowiecki P, Jackowska T. Air Pollutants’ Concentrations Are Associated with Increased Number of RSV Hospitalizations in Polish Children. *J Clin Med*. 2021;10(15):3224. doi:10.3390/jcm10153224

33. Milani GP, Cafora M, Favero C, et al. PM2 .5, PM10 and bronchiolitis severity: A cohort study. *Pediatr Allergy Immunol*. 2022;33(10):e13853. doi:10.1111/pai.13853

34. Chen PC, Mou CH, Chen CW, et al. Roles of Ambient Temperature and PM2.5 on Childhood Acute Bronchitis and Bronchiolitis from Viral Infection. *Viruses*. 2022;14(9):1932. doi:10.3390/v14091932

35. Gallo E, Bressan S, Baraldo S, et al. Increased risk of emergency department presentations for bronchiolitis in infants exposed to air pollution. *Risk Anal*. Published online August 21, 2022. doi:10.1111/risa.14007

36. Xiao D, Guo W, Xu D, Chen J, Liang Z, Zhang X. Three Exposure Metrics for Fine Particulate Matter Associated With Outpatient Visits for Acute Lower Respiratory Infection Among Children in Guangzhou, China. *Front Public Health*. 2022;10:876496. doi:10.3389/fpubh.2022.876496

37. Liang Z, You C, Zhang X, et al. Three exposure metrics of size-specific particulate matter associated with acute lower respiratory infection hospitalization in children: A multi-city time-series analysis in China. *Sci Total Environ*. 2022;816:151636. doi:10.1016/j.scitotenv.2021.151636

38. Dondi A, Manieri E, Betti L, et al. Exposure to outdoor air pollution and risk of hospitalization for bronchiolitis in an urban environment: A 9-year observational study. *Pediatr Pulmonol*. 2023;58(10):2786-2794. doi:10.1002/ppul.26583

39. Wang D, Wang Y, Liu Q, et al. Association of Air Pollution with the Number of Common Respiratory Visits in Children in a Heavily Polluted Central City, China. *Toxics*. 2023;11(10):815. doi:10.3390/toxics11100815

40. Zhang W, Ling J, Zhang R, et al. Short-term effects of air pollution on hospitalization for acute lower respiratory infections in children: a time-series analysis study from Lanzhou, China. *BMC Public Health*. 2023;23(1):1629. doi:10.1186/s12889-023-16533-7

41. Villamil-Osorio M, Moyano-Ariza LF, Camacho-Moreno G, Restrepo-Gualteros SM, Sossa-Briceño MP, Rodriguez-Martinez CE. Multilevel analysis identifying the factors associated with RSV detection in infants admitted for viral bronchiolitis in the era of the COVID-19 pandemic. *Pediatr Pulmonol*. 2023;58(10):2795-2803. doi:10.1002/ppul.26590

42. Lei J, Chen R, Liu C, et al. Fine and coarse particulate air pollution and hospital admissions for a wide range of respiratory diseases: a nationwide case-crossover study. *Int J Epidemiol*. 2023;52(3):715-726. doi:10.1093/ije/dyad056

43. Van Brusselen D, De Troeyer K, van Vliet MP, et al. Air pollution and bronchiolitis: a case-control study in Antwerp, Belgium. *Eur J Pediatr*. 2024;183(5):2431-2442. doi:10.1007/s00431-024-05493-8
